# Biodiversity hotspot assessment in the Altai Mountains transboundary region based on Mammals and Aves

**DOI:** 10.1371/journal.pone.0314075

**Published:** 2024-12-04

**Authors:** Mengqi Yuan, Fang Han, Yue Yang, Aleksandr Dunets, Mikhail Shishin, Ordenbek Mazbayev, Bayarkhuu Batbayar

**Affiliations:** 1 State Key Laboratory of Desert and Oasis Ecology, Key Laboratory of Ecological Safety and Sustainable Development in Arid Lands, Xinjiang Institute of Ecology and Geography, Chinese Academy of Sciences, Urumqi, China; 2 University of Chinese Academy of Sciences, Beijing, China; 3 Altai State University, Barnaul, Russian; 4 Altai State Technical University, Barnaul, Russian; 5 L.N. Gumilyov Eurasian National University, Astana, Kazakhstan; 6 Western Regional School of National University of Mongolia, Khovd, Mongolia; Quaid-i-Azam University Islamabad: Quaid-i-Azam University, PAKISTAN

## Abstract

Most of the world’s mountains are distributed across national boundaries. However, due to the sovereignty of national boundaries, conservation plans between neighboring countries are often uncoordinated. Against the backdrop of impending environmental changes, transboundary mountain ecosystems and biodiversity face significant threats. This study employs the MaxEnt model, leveraging data on climate, topography, landscape, and human activities to predict potential distribution areas for mammals and birds, aiming to identify biodiversity hotspots (BHs) and analyze their distribution mechanisms in the Altai Mountains transboundary region (AMTR). Results indicate that BHs are primarily located near the Russian-Mongolian border, significantly influenced by climate variables, elevation, and human activities. The study also highlights changes in key habitat types (KHTs), particularly transitions between grassland and bareland, and the impact of climate-driven land cover change on the distribution of BHs. Furthermore, the research evaluates the coverage of protected areas and emphasizes the importance of identifying key biodiversity areas (KBAs) and establishing transboundary corridors for enhanced species protection and future environmental change adaptation. The findings underscore the necessity of transboundary cooperation and focused strategies for biodiversity conservation to mitigate the adverse effects of climate change and human activities.

## Introduction

Mountains are global biodiversity hotspots. The complex and diverse habitat types of mountains have given birth to numerous unique and rare animal and plant species, making them a gene pool for the survival and reproduction of various biological species [1, 2], however, the global mountains are mostly distributed across national boundaries spanning the territories of two or more countries [3, 4]. Land borders as an economic, political and symbolic resource [5]. Since the 1990s, more than 40 multifaceted geo-cooperation mechanisms have been established between Mainland Southeast Asia (MSEA) and its neighboring countries (e.g., China and India), turning MSEA into one of the most promising economies in the world [6]. The main purpose of these cooperation mechanisms is to promote peace, stability, economic prosperity and mutual cooperation in the region. The socioeconomic development of border areas has been significantly improved [7, 8]. But with global regional cooperation, geopolitical relations have taken on a new pattern, which has also brought about rapid and extensive boundary land use and cover change (LULCC) [9–11]. National boundaries areas have the same or similar ecological and geographical structure, but show different LULCC trends based on different sovereign jurisdictions [11]. In addition, transboundary landscapes often overlap with BHs, contain critically important ecosystems and provide critical habitat for threatened species.

Mountain transboundary region often harbor rich biodiversity, but at the same time, the protection of transboundary species also faces huge challenges [12–14]. With the impact of global climate change, there will be significant impacts on the ecosystems of various regions [15, 16], which is considered one of the major threats to biodiversity and ecosystems [17, 18]. Natural factors such as climate change may exacerbate ecological risks by changing habitat environments and causing more frequent natural disasters [19–21]. Existing research shows that animals will change their habitat and migrate when exposed to external threats [22–24]. The pessimism is that conservation actions are highly dependent on sovereignty demarcated by national borders, and conservation planning in neighboring countries is often uncoordinated [12], even more pessimistically, due to border control (such as setting up border walls, etc.), artificially separating animal habitats and migration corridors, causing ecological tragedy [25], therefore scientifically identify BHs distribution and migration corridors, and cooperate to promote key ecological source areas (KBAs) protection and construction of transboundary corridors are the common responsibilities of transboundary regions.

The Altai Mountains transboundary region (AMTR) located in Central Asia and spanning China, Mongolia, Kazakhstan, and Russia, is a transnational region with diverse ecosystems ranging from alpine meadows and coniferous forests to pristine rivers and rugged [26]. The Altai Mountains, located in the middle of the Asian continent and spanning China, Mongolia, Kazakhstan, and Russia, are a prime example of such biodiversity richness. This region hosts a wide variety of flora and fauna, including endemic and globally threatened species. Vegetation in the Altai Mountains ranges from coniferous and deciduous broad-leaved forests to alpine meadows and shrublands, supporting species such as Siberian fir, Siberian larch, birch, Mongolian oak, and various endemic plants like Altai Daphne and Altai Lithospermum. Fauna includes iconic species such as the snow leopard, Altai maral, lynx, and a diverse array of bird species including the Altai woodpecker and golden eagle. It has been included in the "Global 200" priority protected ecological areas, also hosts many endemic and globally threatened species [27, 28]. However, transboundary cooperation mechanisms and actions in this region are obviously insufficient. In AMTR, socioeconomic development depends on the utilization of natural resources. Due to the poverty of local people and the expected (international) interest in the natural resources, industrial and infrastructure projects will face increased pressure. In addition, ineffective pasture management, climate change, etc. have caused LULCC, and poaching, border walls, etc. have become major threats to wildlife. Although these four countries have established many protected areas in AMTR, protection measures need to be strengthened. To protect fragmented habitats, especially measuring the impacts of anthropogenic activities and climate change [29, 30], assessment and optimization of protected areas. The diverse ecosystem of AMTR supports a wide range of species, making it an important global biodiversity conservation area.

We focused on mammals and aves in this study. Mammals and birds are not only key indicators of ecosystem health but also play significant roles in their respective habitats. Mammals, such as the snow leopard and Altai maral, are crucial for maintaining the ecological balance by regulating prey populations and facilitating nutrient cycling. Birds, on the other hand, contribute to pollination, seed dispersal, and pest control. Furthermore, these taxa are relatively well-documented in the Global Biodiversity Information Facility (GBIF) database, allowing for robust data analysis using the MaxEnt model. By studying these two groups, we aim to provide a comprehensive understanding of biodiversity distribution patterns and their underlying mechanisms in the AMTR. The distribution of BHs from the perspective of AMTR has not yet been fully understood. Understanding the distribution of biodiversity hotspots in transboundary areas is critical to protecting the unique wildlife in these areas, and also contributes to global biodiversity conservation efforts. Moreover, how future environmental changes will affect the region’s ecosystem, such as the altitude range of species in alpine and subalpine habitats, is critical to predicting future ecological trends.

In this study, we aimed to evaluate comprehensively the distribution and action mechanisms of biodiversity potential hotspots of AMTR and to connect their interrelationships. Based on multiple types of environmental data, the MaxEnt models was used to evaluate biodiversity distribution. This information can provide information for environmental change mitigation and adaptation strategies in the region. While the study focused on AMTR, the findings have broader implications. This study provides useful methods and strategies for biodiversity conservation and management.

## Materials and methods

### Study area

**Fig 1:**
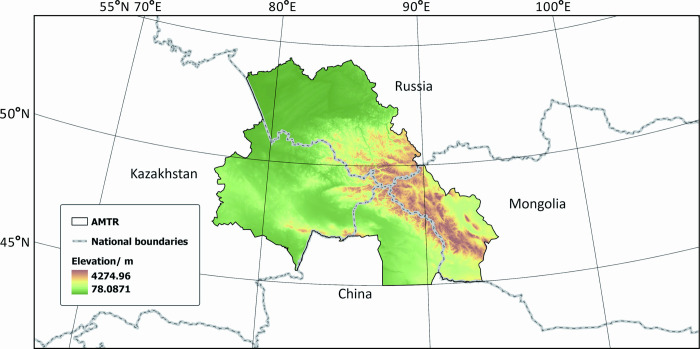
The study area. Note: the Chinese Altai region (CAR), the Kazakhstan Altai region (KAR)the Mongolia Altai region (MAR), the Russian Altai region (RAR). Map Approval Number: GS(2020)4619.

The Altai Mountains are located in the middle of the Asian continent, running diagonally from northwest to southeast across four countries: China, Kazakhstan, Russia, and Mongolia, with a national boundary of about 1,205 km (Fig 1). The study area AMTR includes 7 administrative regions in four countries: China’s Xinjiang Uyghur Autonomous Region Altay region, Russia’s Altai Republic and Altai Krai, Kazakhstan’s East Kazakhstan Oblast, and Abai Oblast, and Mongolia’s Bayan-Ulegi Province and Hovd Provinces, covering a total area of approximately 780,000 km^2^. This area is also the origin and biodiversity center of various rare flora in North and Central Asia. It is the most representative and best-preserved area of the Siberian ecosystem. It has a typical continental climate and unique ecosystem diversity [26]. The Altai Mountain region boasts a rich diversity of flora and fauna, with vegetation primarily comprising coniferous forests, deciduous broad-leaved forests, meadows, shrublands, and alpine plant communities. The coniferous forests are dominated by Siberian fir, Siberian larch, and Siberian pine, forming extensive areas of pristine forest. The deciduous broad-leaved forests, consisting of species such as birch, Mongolian oak, and linden, are distributed in low-altitude areas. In high-altitude regions, endemic plants such as Altai Daphne and Altai Lithospermum thrive. Regarding fauna, the Altai Mountains serve as a crucial habitat for the snow leopard and are home to other mammals such as the Altai maral and lynx, as well as birds like the Altai woodpecker and golden eagle. These species collectively contribute to the complex and diverse ecosystem of the Altai Mountains, highlighting its unique ecological value and the importance of its conservation [31].

### Data sources

This study used data at four levels: climate, topography, landscape, and human activities (Table 1). (1) We downloaded seven environmental climate variables and 19 bioclimatic variables for current climate conditions from the WorldClim dataset (www.worldclim.org). These variables describe temperature, precipitation, annual and seasonal trends in solar radiation, etc., and allow full characterization of species bioclimatic niches with a resolution of approximately 1 km. (2) Digital elevation model (DEM) data (15 arc-second) are from the British Oceanographic Data Centre (BODC) (https://www.gebco.net/data_and_products/gridded_bathymetry_data/). (3) LULC data is released by the National Geomatics Center of China GlobeLand30 data service site (DOI: 10.11769, http://www.globallandcover.com/), and its 2000 and 2020 data are selected, with a resolution of about 30m. (4) The nighttime light data comes from the Earth Observation Group (EOG) (https://eogdata.mines.edu/products/vnl/), 2000 and 2020 is selected, and the resolution is about 450m. (5) Traffic roads in the area The data is provided by OpenStreetMap (OSM) (https://openmaptiles.org/). (6) Population density data is selected from the 2000 and 2020 data of OAK RIDGE NATIONAL NABORATORY (https://landscan.ornl.gov/). The resolution is about 1km. (7) Protected area data comes from Protected Areas (WDPA, https://www.protectedplanet.net/), updated in 2023. By resampling all datasets to a uniform resolution of about 1.3 km^2^, we ensured that all data layers were compatible for integration and analysis in the MaxEnt model. This approach minimizes potential biases and inconsistencies that could arise from using data at different resolutions.

**Table 1:** Data type and source.

Data type	Data	Resolution	Data source
**Climate**	Temperatur	1km	www.worldclim.org
	precipitation		
	wind speed		
	solar radiation		
	BIO1~BIO 19		
**Topography**	Elevation	15 arc-second	https://www.gebco.net/
**Landscape**	Land use and land cover	30m	http://www.globallandcover.com/
**Human activity**	Night light	450m	https://eogdata.mines.edu/products/vnl/
	Population density	1km	https://landscan.ornl.gov/
	Road	30m	https://landscan.ornl.gov/

### Model and operation

We used the MaxEnt model [32] to predict the potential distribution area of species and generate biodiversity hotspots maps. This method has been widely used and verified [33, 34]. The MaxEnt model not only has high accuracy in predicting the distribution of species’ suitable areas, but can also effectively explain the mechanism of various environmental variables on species distribution, and make scientific predictions for timely intervention in future changes of species [34].

The species occurrence data in this study come from the Global Biodiversity Information Facility (GBIF; http://www.gbif.org). Considering species representation, data availability, and species diversity, we chose mammals and birds for our study. The selected species include those classified as CR, EN, VU, and NT on the IUCN Red List, with NT included to monitor species that may soon be at higher risk if not given timely attention. Detailed IUCN classification criteria are shown in S Table 4 in S1 File.

In terms of time, we simulated the current scenario. For environmental variables, we considered the impact of climate, topography, landscape, and human activities. Climate variables, including temperature and precipitation (Table 2), were chosen because they are fundamental drivers of species distributions. These variables define the bioclimatic niches of species and influence their physiological processes, phenology, and habitat suitability. We selected seven environmental climate variables and 19 bioclimatic variables from the WorldClim dataset to capture the seasonal and annual climatic variations that affect the Altai Mountains region. Topography variables such as elevation and slope were included to account for the micro-environmental variations that influence species distributions. Elevation affects temperature and precipitation patterns, while slope influences soil stability and water drainage. These factors are critical in mountainous regions like the Altai Mountains, where altitude can create diverse microhabitats. Landscape variables, including land use and land cover (LULC), were selected to reflect the spatial structure and composition of the habitat. The landscape heterogeneity was measured using Shannon’s Diversity Index (SHDI) to represent habitat diversity, and the landscape contagion index (CONTAG) to depict landscape connectivity. These indices are crucial for understanding habitat fragmentation and connectivity, which are important for species movement and survival [35]. Human activity variables, such as population density, road distance, and night light data, were chosen to represent anthropogenic pressures on the environment. These variables are indicators of human presence and infrastructure development, which can lead to habitat loss, degradation, and fragmentation. Including these variables helps in assessing the impact of human activities on species distributions and identifying areas that require conservation interventions.

**Table 2:** The MaxEnt model uses data.

Driving force	Driving factors	Unit	Resolution	Factorcode
**Climate**	BIO1~BIO 19	° C, mm, etc.	1km	bio1~19
**Topography**	Elevation	m	30m	elev
	Slope	°	30m	slo
**Landscape**	Land use and land cover	-	30m	lulc
	Water resource distance	m	30m	distowater
	Contagion Index	-	30m	contag
	Shannon’s Diversity Index	-	30m	shdi
**Human activity**	Night light	-	450m	ntl
	Population density	km^-2^	1km	peo
	Road distance	m	30m	distoroad

Note: BIO1: Annual Mean Temperature; BIO2: Mean Diurnal Range (Mean of monthly (max temp—min temp)); BIO3: Isothermality (BIO2/BIO7) (×100); BIO4: Temperature Seasonality (standard deviation ×100); BIO5: Max Temperature of Warmest Month; BIO6: Min Temperature of Coldest Month; BIO7: Temperature Annual Range (BIO5-BIO6); BIO8: Mean Temperature of Wettest Quarter; BIO9 = Mean Temperature of Driest Quarter; BIO10: Mean Temperature of Warmest Quarter; BIO11: Mean Temperature of Coldest Quarter; BIO12 = Annual Precipitation; BIO13: Precipitation of Wettest Month; BIO14: Precipitation of Driest Month; BIO15: Precipitation Seasonality (Coefficient of Variation); BIO16: Precipitation of Wettest Quarter; BIO17: Precipitation of Driest Quarter; BIO18: Precipitation of Warmest Quarter; BIO19: Precipitation of Coldest Quarter.

**Fig 2:**
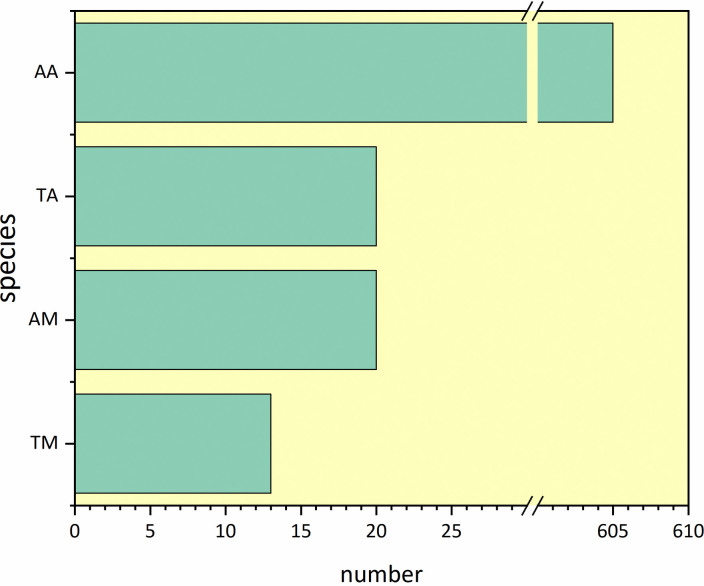
Species classification and operational numbers in MaxEnt models. TM: threatened Mammals; AM: all Mammals; TA: threatened Aves; AA: all Aves.

Bioclimatic (BIO1~BIO19) variables were collinearly eliminated using Band Collection Statistic in ArcGIS 10.8 (selection range -0.7~0.7). We selected a certain number of species occurrence data and used the built-in tool [Spatially Rarefy Occurrence Data for SDMs (reduce spatial autocorrelation)] of SDM Toolbox v2.5 (http://www.sdmtoolbox.org/) to reduce the resolution to rarefy. The data frame was set to 1 km to avoid spatial autocorrelation caused by species distribution points being too close, which could affect the accuracy of prediction results [36, 37]. Finally, we eliminated the data with AUC less than 0.8 and obtained a total of 20 species of Mammals (Detailed procedures are provided in S1 File), including 7 threatened species, such as Snow leopard (*Panthera uncia*), Siberian Ibex (*Capra sibirica*), etc., and 13 species of least concern, such as Siberian Chipmunk (*Eutamias sibiricus*), etc. There are 605 species of Aves, including 20 threatened species, such as Cinereous Vulture (*Aegypius monachus*), and 585 non-threatened species, such as Chuckar (*Alectoris chukar*) (Fig 2).

## Results

### Distribution characteristics of BHs

**Fig 3:**
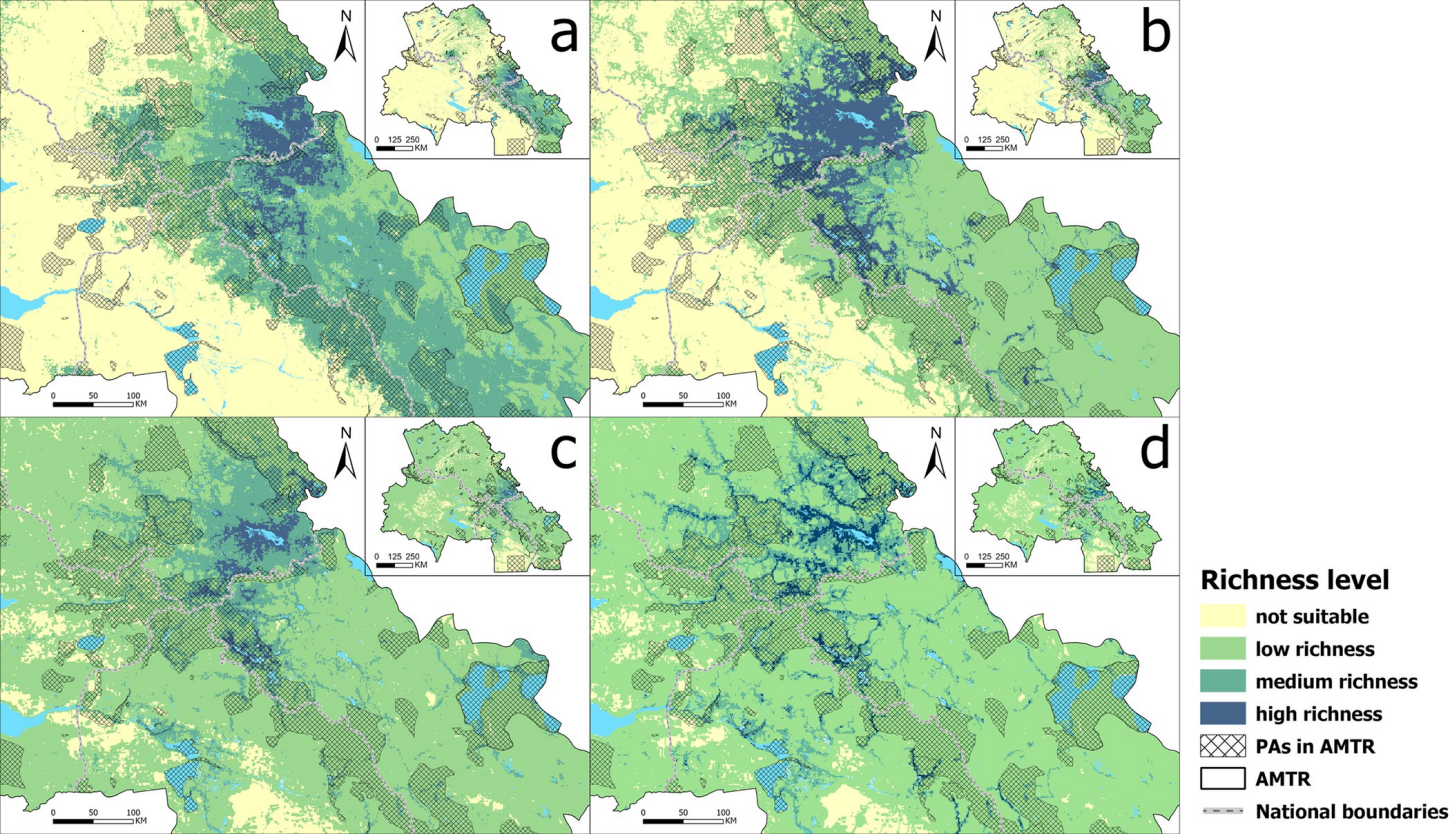
Distribution of biodiversity hotspot. a. TM; b. AM; c. TA; d. AA.

Fig 3 shows the BHs of AMTR Mammals and Aves respectively. There is a certain spatial similarity in BHs between TM and AM species. High richness (HR) is mainly distributed in the Russian-Mongolian border areas and near-border areas of Mongolia. Medium richness (MR) also has obvious distribution characteristics along the National boundaries and covers most of the Altai Mountains. Low richness (LR) is mainly distributed in MAR. From an area perspective, the HR of TM is approximately 9943.29km^2^, the MR is approximately 94589.96km^2^, and the LR is approximately 131667.55km^2^. The HR of AM is approximately 22877.47 km^2^, and the HR is larger than the HR of AM.

The spatial distribution of HR of AA is also similar to AM and is mainly distributed in the Russian-Mongolian border areas and the near-border areas of Mongolia. Similarly, the area of MR (38482.06km^2^) for TA was larger than the area (35976.61km^2^) for AA. The HR (6012.66km^2^) and LR (6918.04km^2^) of TA are smaller than that of AA (HR area: 615955.75km^2^, LR area: 636102.77km^2^), and there is spatial overlap between AA distribution and water sources.

This difference may be due to the fact that most AA have similar suitable habitat areas and there are differences in spatial distribution among AM. From the perspective of each partition, AA diversity is mainly concentrated in the RAR (the HR ratio of TA is 82.40%, and the HR ratio of AA is 83.19%; the MR ratio of TA is 64.30%, and the MR ratio of AA is 72.44%). AM diversity is mainly distributed in the MAR (the HR of TM proportion is 54.84%, the HR of AM proportion is 52.63%; the MR of TM proportion is 59.67%, the MR of AM proportion is 24.01%) and RAR (the HR of TM proportion is 44.98%, the HR ratio of AM is 37.11%; the MR ratio of TM is 24.39%, and the MR ratio of AM is 65.73%).

In summary, the BHs of Aves are more widely distributed than Mammals in terms of overall richness, but when focusing on HR and MR, Mammals are wider. From a geographical perspective, the hotspots of species diversity demonstrate distinct patterns along national boundaries. Furthermore, the distribution of Mammals is characterized by clustering, whereas distribution of Aves displays a linear pattern, primarily attributed to their alignment along water sources and river valleys. Notably, BHs are predominantly situated in the northern region of AMTR, highlighting significant regional disparities.

### Mechanism of BHs distribution

**Fig 4:**
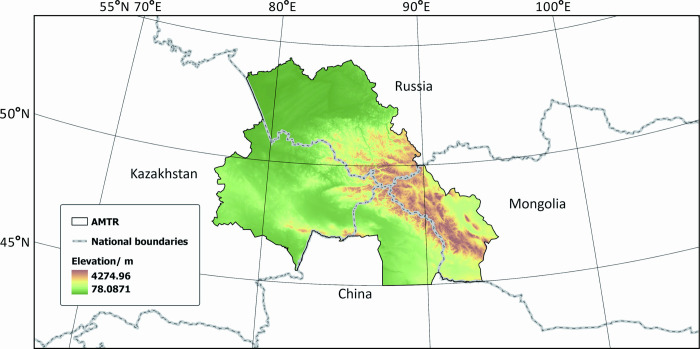
Mechanism of action of biodiversity hotspots. The orange dots are averages; a. TM; b. AM; c. TA; d. AA.

Fig 4 shows the contribution of influencing factors that affect the distribution of BHs for TM, AM, TA, and AA. bio19 (26.44%), elev (15.32%), and bio11 (13.84%) have major contributions to the distribution of BHs of TM. The main contributing factors to the distribution of BHs of AM are bio11 (29.53%), distoraod (17.20%), elev (12.26%), and bio19 (10.80%). Factors such as bio11 (36.21%), peo (11.72%), and distoraod (11.41%) have a high contribution to the distribution of BHs of TA. The main contributing factors to the distribution of BHs of AA, include: bio11 (21.79%), distoraod (19.52%), waterdistance (11.48%), peo (10.86%). We can see that climate factors are still the key factors that dominate species BHs. BHs for TM, AM, TA and AA are mainly driven by climate, where the climate contributions to TM, AM and TA are 66.95%, 59.65% and 52.04% respectively (sum of climate factors). In addition, the climate contribution of threatened animals is higher than that of non-threatened animals, such as TM>AM, TA>AA. This could mean that the effects of climate change are more severe for species that already face existential threats. Aves, on the other hand, are more dependent on human activities and landscapes than Mammals, and are especially more dependent on human activities than on other factors. This dependence may lead to more severe impacts of human activities on Aves.

## Discussion

### Climate-driven land cover change and biodiversity hotspot distribution mechanism

**Fig 5:**
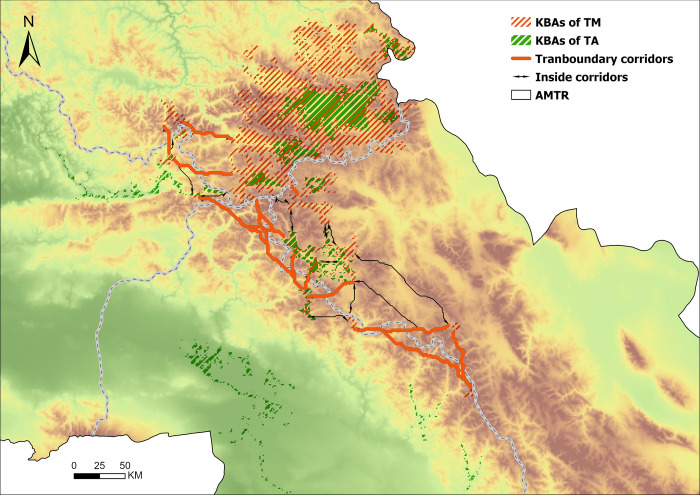
Driving factor of LULCC. a. driving factor contribution; b. the dominant role of different driving force on LULCC.

Between the years 2000 and 2020, notable changes in LULC occurred within AMTR. Among these changes, the most prominent was the conversion of approximately 49,262.99 km^2^ of grassland to bareland, constituting 38.31% of the total change in area. Subsequently, there was a significant transformation of approximately 19,611.74 km^2^ from grassland to forest, accounting for 15.25% of the total change (See S2 for detailed analysis in S1 File). The LEAS module in the PLUS model (see S3 in S1 File) was used to analyze the driving factors of LULCC in AMTR and each subregion from 2000 to 2020. The results are shown in Fig 5. Over the past 20 years, climate has dominated the overall LULCC (50.87%; we define more than 20% of the second influencing factors as dominant). From the perspective of each LULC type, climate has dominated changes in forest (56.26%), grassland (62.31%), bareland (74.43%), and snow/ice (60.38%). Human activities (56.18%) dominate the changes in artificial surfaces, and changes in cropland are mainly caused by human activities (48.08%) and climate (35.04%) playing a joint role. Wetland changes are mainly driven by climate (52.82%) and topography (40.95%). Water is similar to wetlands, with climate and topography contributing 45.34% and 48.43%, respectively.

The dominance of climate as a driving factor for LULCC and biodiversity hotspot distribution suggests that species in the AMTR may face greater challenges due to future climate change. This is because a strong single dominance increases the likelihood of species becoming extinct due to changes in future dominant factors [38]. These findings underscore the need for adaptive management strategies to mitigate the impacts of climate change on biodiversity hotspots.

### Spatial characteristics of BHs and key environmental factors

**Fig 6:**
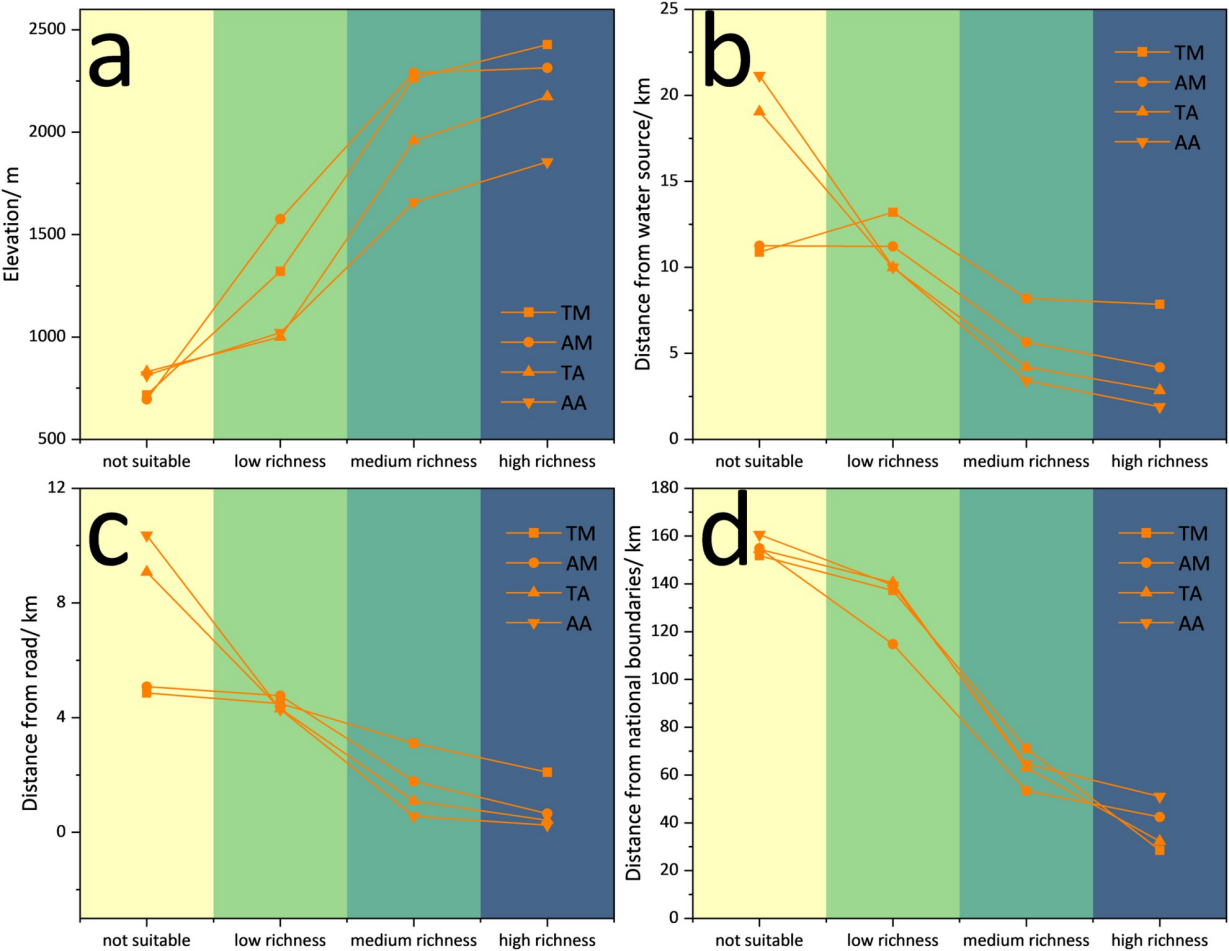
Spatial relationship between species and environmental factors.

Understanding the spatial relationship between BHs and key environmental factors can help us more fully understand the distribution of BHs in AMTR. This understanding not only helps reveal species distribution patterns but also provides a scientific basis for ecological management and conservation to better protect species diversity [33, 39, 40]. In our study, we analyzed the relationship between species richness and environmental factors in TM, AM, AA, and TA (Fig 6). We observed a significant positive correlation between species richness and elevation, and a clear negative correlation between distance from national borders, water sources, and roads. This means that areas with high species diversity are more likely to be located at high altitudes and close to national boundaries, roads, and water sources.

**Fig 7:**
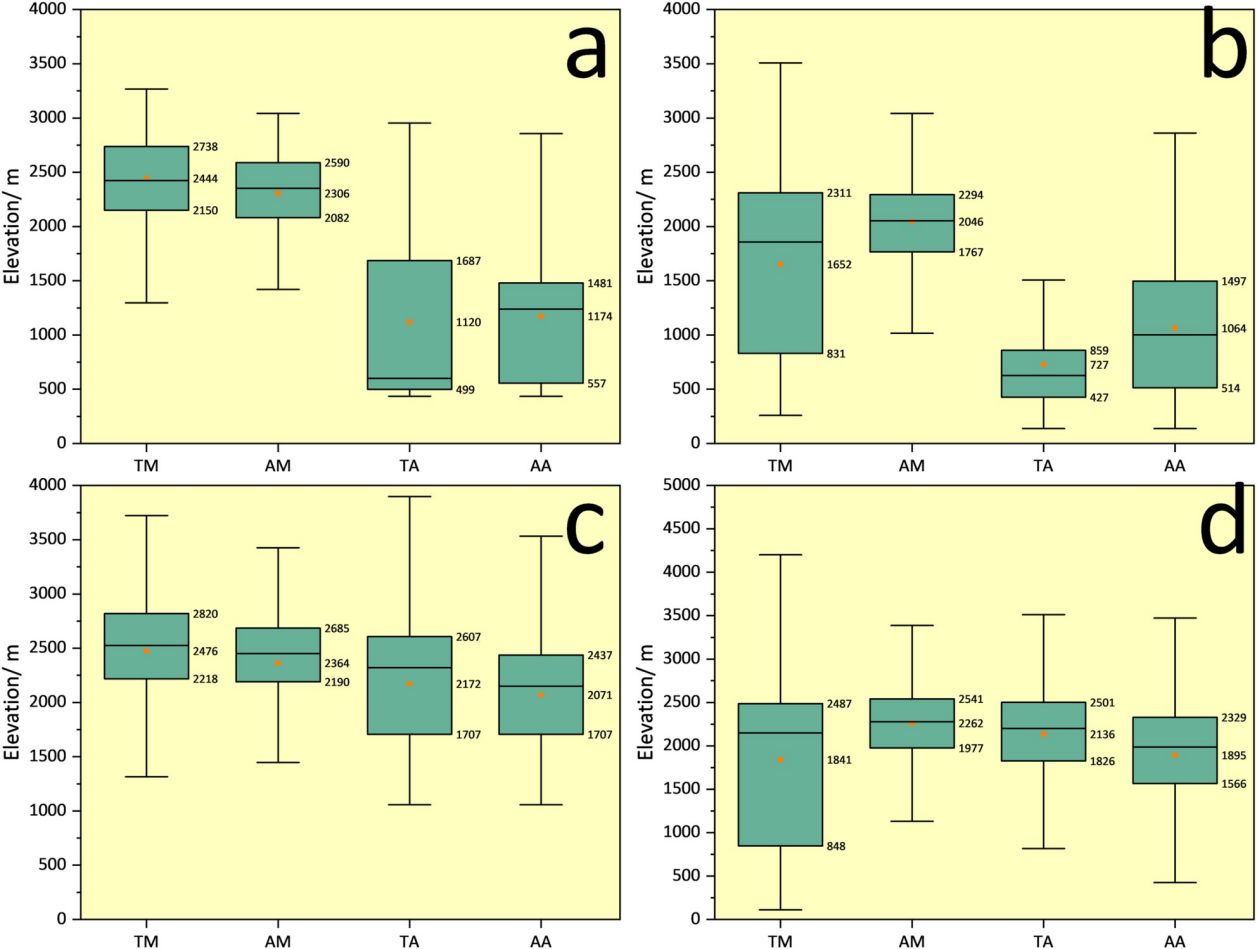
Elevation distribution of biodiversity hotspots. a. CAR; b. KAR; c. MAR; d. RAR.

These spatial relationships highlight the influence of topography, human activities, and landscape on biodiversity. Specifically, in terms of distance to water sources, aves are closer to water sources than mammals; in terms of distance from roads, most aves are closer to roads than mammals, which may reflect their differing habitat preferences [41, 42]. Regarding altitude distribution, mammals are generally distributed at higher altitudes than aves, and threatened species are found at even higher altitudes. We further analyzed the vertical distribution of species in each subregion (Fig 7) to clarify the spatial relationship of topography, a highly contributing element. The large altitude distribution difference between mammals and aves is mainly attributed to the spatial distribution differences in CAR and KAR, where mammals are primarily distributed in higher mountains, and aves in valleys or river valleys with lower altitudes.

These findings provide useful information for ecological management and conservation. Considering the relationship between species distribution and human activities, we can develop more targeted conservation strategies to mitigate potential threats to threatened species. For example, countries could jointly manage high-altitude areas along national boundaries and focus on the impact of water source protection and transportation on animals.

### Key habitat types of BHs

The identification of key habitat types (KHTs) is crucial to understanding the distribution and living conditions of biodiversity hotspots [43]. Different species have different adaptability to various habitat types, and changes in habitat types will lead to changes in species distribution ranges and the unsustainable maintenance of habitats [44, 45]. By identifying KHTs, we can better understand the ecological needs and adaptive capabilities of species and thus formulate targeted conservation measures to maintain and restore the species’ living environment.

**Fig 8:**
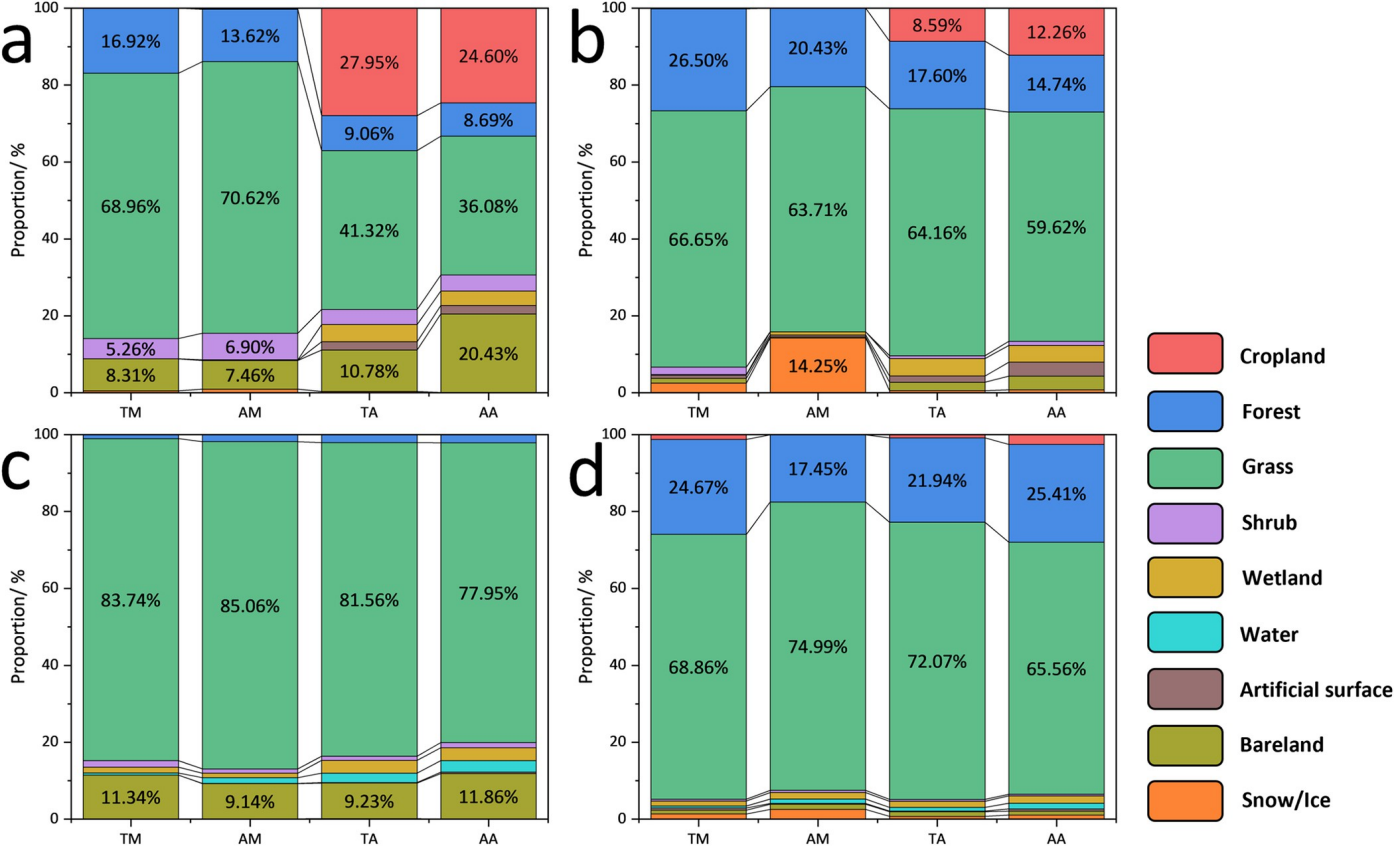
Proportion of habitat types. a. CAR; b. KAR; c. MAR; d. RAR.

Our analysis of the LULC data with HR and MR in each region shows that the KHTs have similar characteristics (Fig 8). Grassland accounts for the majority of KHTs for the four species in AMTR, and forest is the second most important KHT for TM and AM in addition to MAR, and for TA and AA in KAR. Additionally, cropland has a larger proportion in the habitats of TA and AA in CAR and KAR, while bareland also has a significant proportion in the habitats of each species in CAR and MAR.

Grassland are critical habitats for many mammal species such as the Siberian Ibex and the Altai Maral, which rely on open grassland for grazing and avoiding predators. Forests provide crucial habitats for species like the Snow Leopard and the Altai Lynx, offering cover and prey availability. Forests also support a variety of bird species, including the Siberian Jay and the Great Grey Owl, which rely on dense forest habitats for nesting and foraging. Although cropland is primarily an anthropogenic habitat, some species have adapted to these environments. For instance, birds like the Lapland Longspur can often be found in agricultural areas, exploiting the open fields for feeding. Bareland areas, though less biodiverse, provide essential habitats for certain specialized species. For example, the Pallas’s Pika prefers rocky, barren areas where it can find shelter and food.

Overall, CAR and KAR have richer habitat types than MAR and RAR, and aves have richer and more balanced habitat types than mammals. The previous results show that past climate dominated LULCC, with significant changes in grassland and forest KHTs. Although the landscape layer does not have the highest contribution to the distribution of BHs, it is foreseeable that under the combined effects of climate change and landscape change, species will inevitably be affected to some extent [46, 47]. This highlights the need for continuous monitoring and adaptive management to ensure the sustainability of these key habitats.

### Evaluation of protected areas coverage

**Fig 9:**
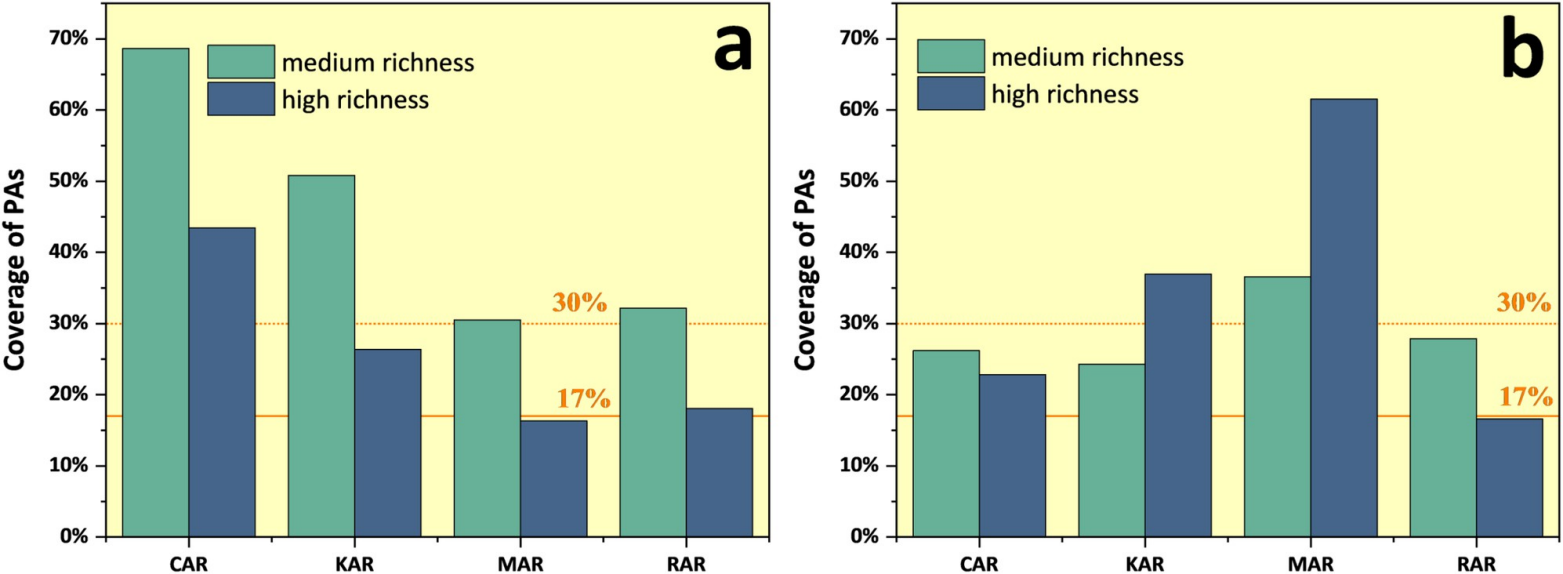
Protected area coverage of threatened species hotspots. a. TM; b. TA.

Countries in the Altai region have established numerous protected areas (PAs) at different levels within the region to protect natural landscapes and biodiversity. The coverage of RH and MR of TM and TA in PAs is shown in Fig 9. Only the RH of TM in MAR and the RH of TA in RAR failed to reach the Aichi target (17% by 2020) [48], indicating that countries in the region have made positive strides in protecting biodiversity over the past few decades.

Looking forward, the 2020 Global Biodiversity Framework (GBF) aims to expand the coverage of well-connected protected areas and other effective regional conservation measures to 30% by 2030 [49]. Optimistically, the PAs coverage of MR of TM and HR of KAR, MR, and HR of MAR has already reached 30%, while the rest are less than 30%. This indicates that there is still room for regional parties to improve their efforts in AMTR to better cope with future environmental changes.

To enhance the effectiveness of protected areas and expand their coverage, several strategies can be implemented. Enhancing connectivity by establishing ecological corridors between existing PAs is crucial for facilitating species migration and genetic exchange. This can be achieved by identifying and protecting key migration routes, especially those connecting transboundary areas [50]. Strengthening legal frameworks is also essential, which involves implementing stronger legal protections for PAs and enhancing enforcement mechanisms to prevent illegal activities such as poaching and habitat destruction.

Another critical strategy is community involvement. Engaging local communities in conservation efforts through education and providing incentives for sustainable land use practices can help in reducing human-wildlife conflicts and promoting coexistence. Regular monitoring and research are also vital. Regular monitoring of biodiversity and ecological conditions within PAs can inform adaptive management strategies. Investing in research to understand the impacts of climate change and human activities on biodiversity can provide valuable insights for effective conservation planning.

Securing adequate funding and resources is fundamental for the management and expansion of PAs. This includes leveraging international funding mechanisms and partnerships to mobilize the necessary resources for these efforts. By implementing these strategies, regional parties can improve their conservation efforts in AMTR and better cope with future environmental changes, thereby ensuring the resilience and sustainability of this ecologically significant region.

### Identification of key biodiversity areas and transboundary corridors

**Fig 10:**
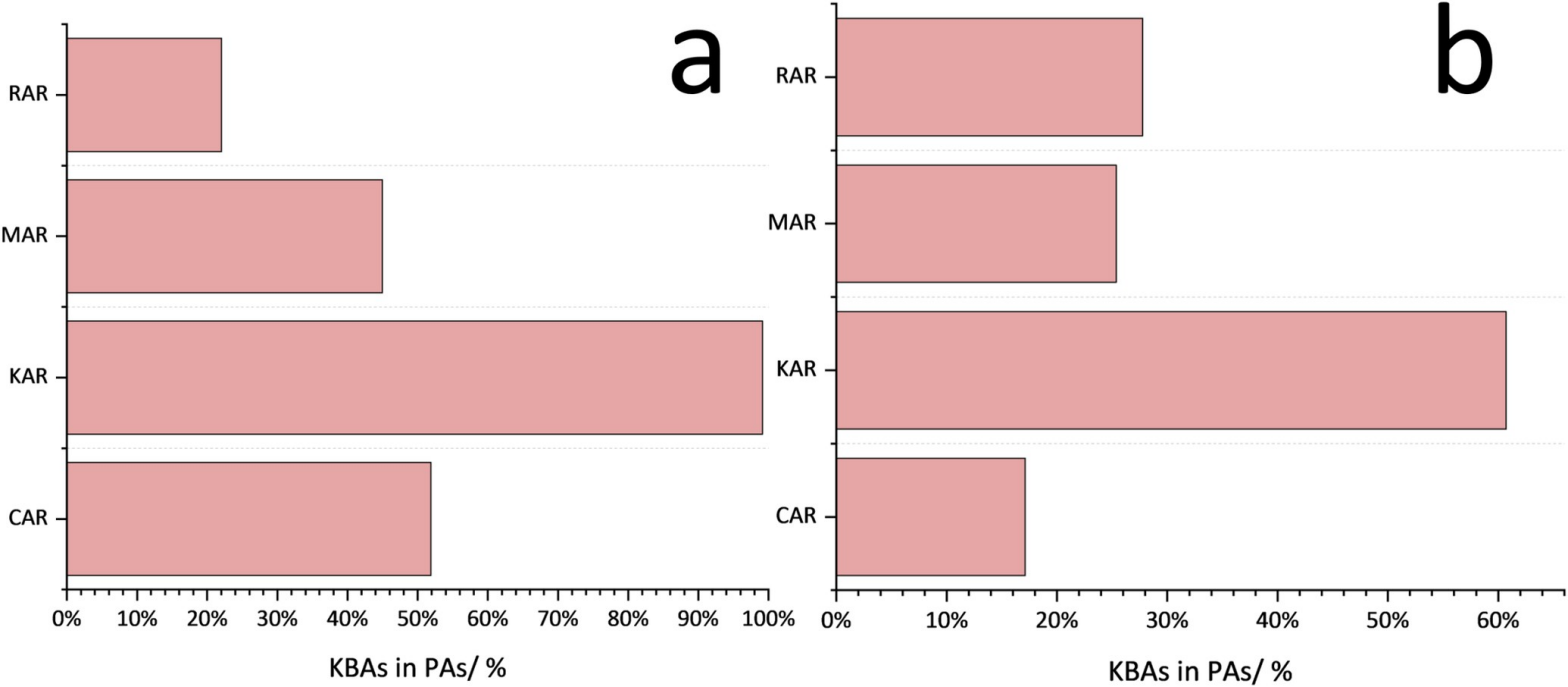
Protected area coverage of KBAs in each region.

In order to further promote the protection of threatened species and cope with future environmental changes, it is essential to identify key biodiversity areas (KBAs) and transboundary corridors in various regions and promote transboundary protection [51–53]. We reclassified the interior from the perspective of each region (by 0~20%, 20%~50%, >50% within the region) to determine the richness level within each region and better identify the KBAs and key PAs. Areas with high richness (HR) in each region are considered priorities for biodiversity conservation [34, 54] (S2 Table in S1 File). This classification provides a reference for relevant parties to focus their protection efforts. We found that most KBAs and protected areas have good coverage of KABs of threatened mammals (TM) (>30%), but the protected areas (PAs) coverage of KBAs of TM in RAR is only 22.04%. For the PAs coverage of KBAs of threatened aves (TA), only KAR exceeds 30% (Fig 10). This indicates that there is still room for improving protected area settings in each district in the future.

**Fig 11:**
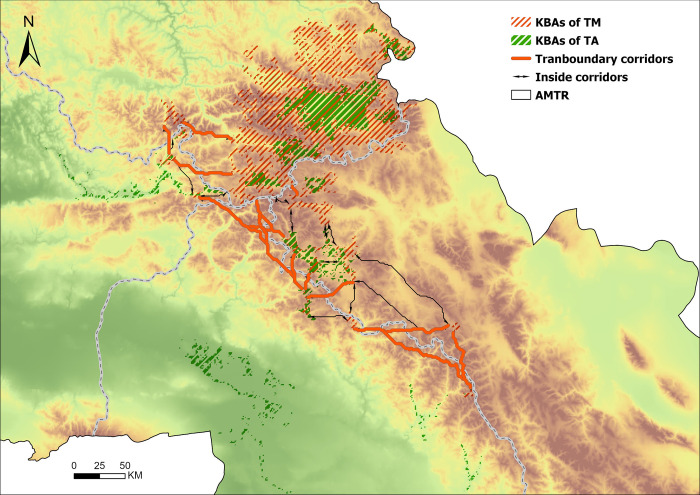
The ecological source and the corridor in AMTR.

Since mammals, especially large mammals, often undergo long-distance migrations [55, 56], transboundary migration often occurs in mountainous regions and is affected by human factors such as border walls or physical jurisdiction [50]. Identifying transboundary migration corridors is critical for the conservation of threatened mammals. Therefore, we constructed an ecological resistance surface (S Table 3 in S1 File) based on the TM distribution tendency using five factors such as elevation and landscape, and input the KBAs of TM and resistance into the Linkage Mapper toolbox (https://linkagemapper.org/, see S4 for detailed operation in S1 File). In AMTR, 25 corridors were identified (Fig 11), including 14 transboundary corridors. There are 3 between Russia and Kazakhstan, 1 between Russia and Mongolia, 8 between China and Mongolia, and 2 between Russia and China and Kazakhstan.

The identification of transboundary corridors provides a reference for relevant parties to protect TM, especially by pointing out feasible locations for dismantling unreasonable border walls [28]. Moreover, most transboundary corridors (79.13%) are distributed within protected areas of various countries, which greatly increases the possibility of precise protection. These corridors facilitate the movement of species across borders, ensuring genetic flow and reducing the risk of inbreeding. This is particularly important for large mammals that require extensive territories and connectivity between different habitat patches to maintain viable populations.

Furthermore, these identified areas and corridors contribute to regional conservation strategies by enhancing habitat connectivity, allowing species to migrate in response to environmental changes, and providing a buffer against habitat fragmentation. By maintaining and improving these corridors, we can support genetic diversity and species resilience, which are crucial for adapting to climate change and other environmental pressures. For instance, corridors connecting protected areas can help mitigate the impacts of habitat loss by providing alternative routes for species migration and dispersal, thereby reducing the likelihood of local extinctions. This integrated approach not only benefits individual species but also strengthens the overall ecological network, promoting a more resilient and sustainable ecosystem.

Implementing these strategies at a regional level requires coordinated efforts among the countries involved. This includes harmonizing conservation policies, sharing data and resources, and engaging in joint conservation projects. Transboundary cooperation can enhance the effectiveness of conservation measures by addressing ecological processes that transcend national borders [57]. For example, synchronized monitoring and management activities can lead to better protection of migratory routes and critical habitats, while joint research initiatives can improve our understanding of species’ responses to environmental changes and inform adaptive management practices. These comprehensive analyses and recommendations underscore the critical need for continued and enhanced transboundary cooperation to effectively protect biodiversity in the AMTR. By addressing both current and future challenges, such strategies aim to ensure the resilience and sustainability of this ecologically significant region.

## Conclusion

This study presents a comprehensive analysis of BHs within AMTR, employing a multidimensional dataset that encompasses climate, topography, landscape, and human activities. Our findings highlight the Altai Mountains as a critical region for biodiversity, underscoring the unique ecological significance and conservation value of this area. Through the application of the MaxEnt modeling approach, we were able to predict the potential distribution areas of species, identifying both threatened and non-threatened species across the region.

The results clearly demonstrate that BHs in AMTR exhibit distinct spatial patterns, with significant variability in richness and distribution of species across the four nations. The study identifies that the distribution of mammals and aves is largely influenced by a combination of climatic variables and human activities, with climate change posing a significant threat to these species. The analysis of land use and land cover changes further emphasizes the profound impact of climate-driven alterations on habitat availability and quality.

Moreover, our evaluation of protected areas within the region indicates that, although substantial progress has been made towards biodiversity conservation, further efforts are required to achieve the Global Biodiversity Framework’s target of expanding protected area coverage. The identification of KBAs and transboundary corridors offers a strategic pathway for enhancing conservation efforts, facilitating species migration, and ensuring the long-term sustainability of habitats.

This study underscores the importance of integrating multi-level environmental data and advanced modeling techniques to better understand the complex dynamics of BHs. It also highlights the critical need for transboundary cooperation and concerted conservation strategies to mitigate the impacts of climate change and human activities on biodiversity in the Altai Mountains. Specifically, strategies such as enhancing connectivity through ecological corridors, strengthening legal frameworks, involving local communities, and securing adequate funding are crucial. Future research should focus on refining predictive models, exploring the effectiveness of conservation interventions, and enhancing the connectivity of protected areas to ensure the resilience of biodiversity in this globally significant region. These targeted actions and a cohesive transboundary approach are essential for achieving the study’s aims and ensuring the effective conservation and sustainability of the Altai Mountains’ unique biodiversity.

## Supporting information

S1 File (PDF)
