# Correction: Biodiversity hotspot assessment in the Altai Mountains transboundary region based on Mammals and Aves

**DOI:** 10.1371/journal.pone.0322007

**Published:** 2025-04-04

**Authors:** Mengqi Yuan, Fang Han, Yue Yang, Aleksandr Dunets, Mikhail Shishin, Ordenbek Mazbayev, Bayarkhuu Batbayar

Fig 4 is uploaded incorrectly. Please see the correct Fig 4 here.

**Fig 4:**
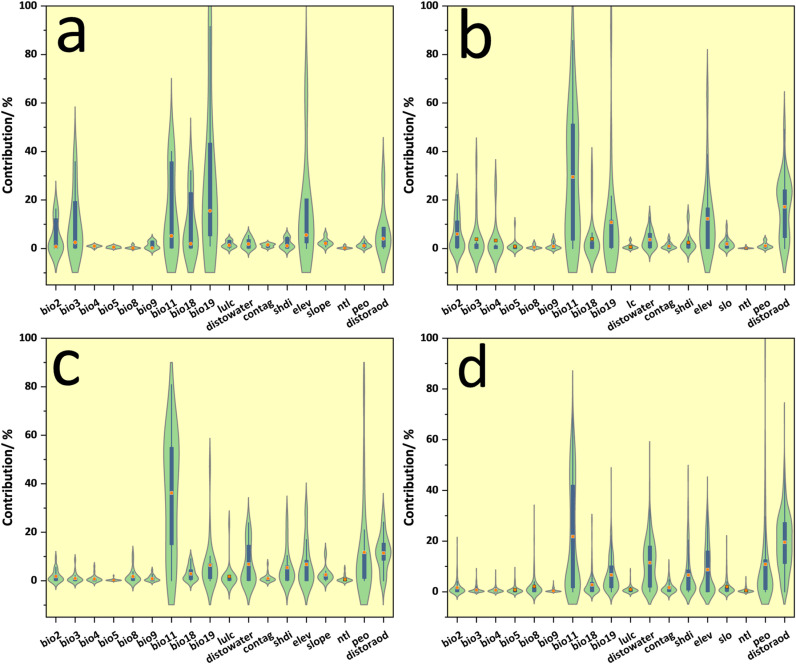
Mechanism of action of biodiversity hotspots.

The orange dots are averages; a. TM; b. AM; c. TA; d. AA.
