# Supplementary material for: Biodiversity hotspot assessment in the Altai Mountains transboundary region based on Mammals and Aves
Source: PLoS One. 2024 Dec 4;19(12):e0314075. doi: 10.1371/journal.pone.0314075 (PMC11616857; doi:10.1371/journal.pone.0314075)
Supplement: S1 File — (PDF) [file pone.0314075.s001.pdf]

Supporting information

S1 Model and Operation

S1.1 Data Preparation

Unification of the coordinate system, range, resolution, and the number of rows and columns of the variable raster layers.

Conversion to ASCII format for compatibility with MaxEnt 3.4.4.

S1.2 Model Operation

Loading species occurrence data and environmental variable raster layers into MaxEnt 3.4.4 (https://biodiversityinformatics.amnh.org/open_source/maxent/).

Training the model with 75% of the distribution points and using the remaining 25% for validation.

Utilizing the automatic feature selection function to avoid over-fitting.

Applying the Jackknife procedure to analyze the relative impact of environmental variables.

Using the area under the curve (AUC) of the receiver operating characteristics curve (ROC) to verify model simulation results.

Converting continuous species potential distribution predictions into binary distributions using the maximum training sensitivity plus specificity threshold.

Setting the maximum number of background points to 10,000 and the maximum number of iterations per run to 1,000 to ensure model convergence.

Performing 10 replicates using repeated split samples to measure variability and averaging the results.

S1.3 Species Selection and Data Filtering

Final selection of species with AUC greater than 0.8.

Inclusion of 20 mammal species, including 7 threatened species such as the Snow Leopard (Panthera uncia) and Siberian Ibex (Capra sibirica), and 13 species of least concern, such as the Siberian Chipmunk (Eutamias sibiricus).

Inclusion of 605 bird species, including 20 threatened species such as the Cinereous Vulture (Aegypius monachus) and 585 non-threatened species such as the Chukar (Alectoris chukar).

S2 Land use and cover changes

Between the years 2000 and 2020, notable changes in LULC occurred within AMTR, as illustrated in Fig. 3. Among these changes, the most prominent was the conversion of approximately 49262.99 km2 of grass to bareland, constituting 38.31% of the total change in area. Subsequently, there was a significant transformation of approximately 19611.74 km2 from grass to forest, accounting for 15.25% of the total change. The alterations in various types of LULC, including transitions from forest to grass, bareland to grass, grass to cropland, cropland to grass, grass to shrub, shrub to

grass, and grass to wetland, each accounted for changes ranging from 1% to 10%. In contrast, the proportions of LULCC for the remaining types were all below 1%.

From the perspective of various regions, the Chinese Altai region (CAR) has changed from grass to bareland in the past 20 years. The area has reached 38540.01km2, accounting for 75.55% of the total changed area, becoming the most important LULCC. Secondly, the area change from bareland to grass is about 3764.03km2, accounting for 7.38% of the total changed area. The proportions of other types of LULCC were less than 5%. LULCC in the Kazakhstan Altai region (KAR) are mainly grass, with 21.88% of the change being from grass to bareland, and 17.41% from grass to cropland. 11.76% is from grass to forest, and the corresponding areas are 6964.01km2, 5541.01km2, and 3742.09km2 respectively. In the Mongolia Altai region (MAR), LULCC were characterized by a conversion of 3438.03 km2 of grass to bareland, constituting 33.67% of the total change. Simultaneously, there was an alteration of 3,833.12 km2 from bare land to grassland, representing 37.54% of the overall change. Types of LULCC accounted for proportions less than 5%. Among LULCC in the Russian Altai region (RAR), the area of change from grass to forest is 14821.01km2, accounting for 41.54%, the area of change from forest to grass is 6904.07km2, accounting for 19.35%, and the area of change from cropland to grass is 6904.07km2, accounting for 19.35%. The changed area was 3643.02km2, accounting for 10.21%. The changed area from grass to cropland was 3318.01km2, accounting for 9.30%. The proportions of other types of LULCC were less than 5%.

Overall, grass of AMTR area decreased by 55834.53km2, bareland area increased by 37008.53km2, forest area increased by 9008.64km2, cropland area increased by 3464.69km2, shrub area increased by 2704.12km2, and wetland area increased by 1805.67km2. In the Chinese Altai region(CAR), the grass and forest areas decreased by 38026.51km2 and 35.17km2 respectively, while the bareland area increased the most, with a total increase of 33252.07km2. In the Kazakhstan Altai region (KAR), grass decreased by 10538.62km2, while bareland increased the most, about 5356.67km2. In addition, the cropland area increased by 2504.71km2 and the artificial surface area increased by 238.37km2, which was the largest increase among the four countries. In the Mongolia Altai region (MAR), bareland and grass decreased by 754.69km2 and 66.90 km2 respectively, wetland area increased by 572.93km2, and ice/snow area increased by 140.60km2, which are the two land types with the largest increase in area in the region. The the Russian Altai region (RAR) grass area decreased by approximately 7202.49km2, followed by the bareland area which decreased by 845.53km2. It is worth noting that the forest area has increased by 7677.04km2, and the ice and snow area has increased by 233.15km2, which are the types with the largest increase in area among the four regions. LULCC show obvious regional differences, and land bareness mainly occurs south of the Altai Mountains.

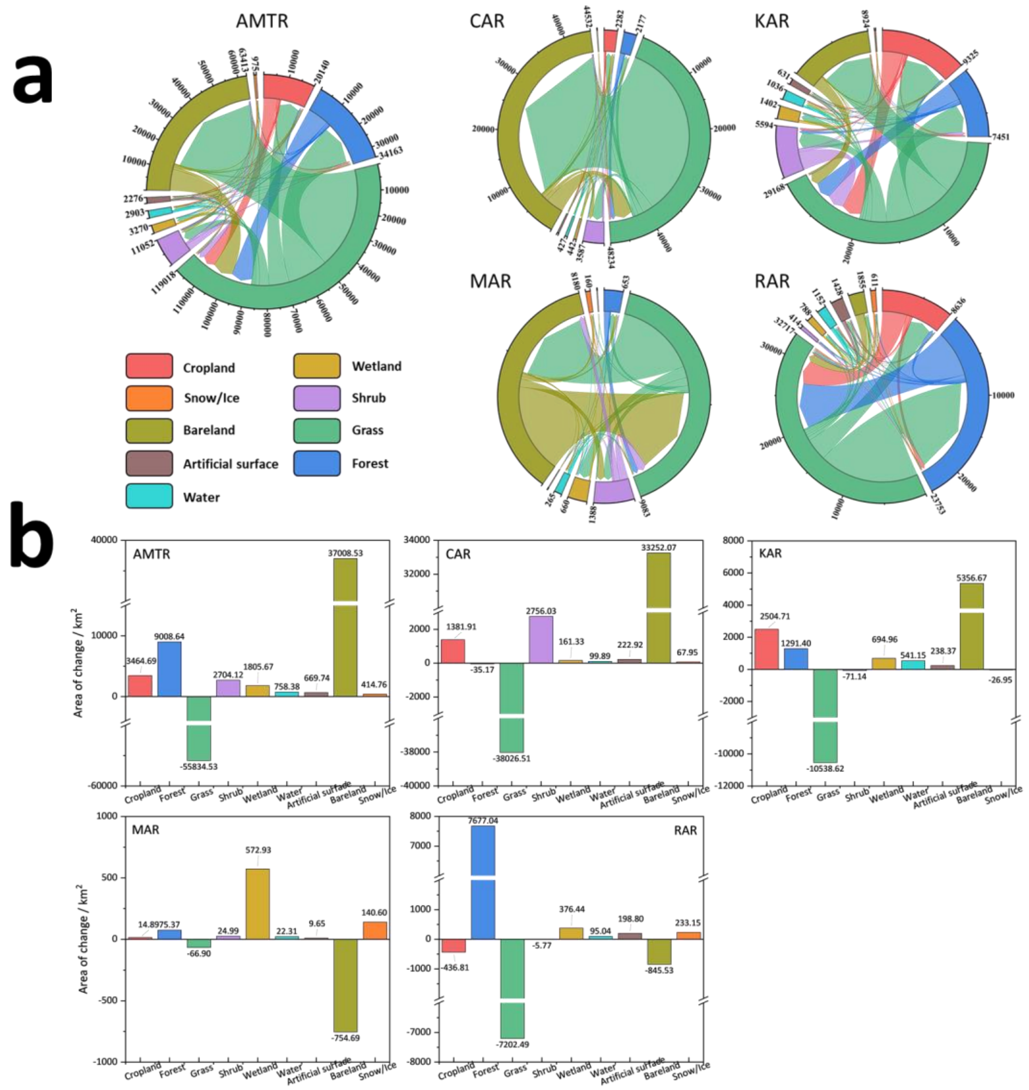

S Fig 1 Transformation and area of LULCC in AMTR (a. Chord diagram of shifts among different land use types between 2000 and 2020. b. Area histogram of LULCC).

S3 PLUS model and operation

Patch-generating Land Use Simulation Model (PLUS) is a future land use change simulation model that integrates a land expansion strategy analysis module and a cellular automaton model based on multiple types of random patch seeds. The land expansion strategy analysis module (land expansion analysis strategy, LEAS) extracts the expansion of various land uses between the two periods of LULCC and uses the random forest algorithm to mine the factors of expansion and driving forces of various land uses one by one to obtain the development probabilities and driving forces of various land uses. The contribution of factors to the expansion of various land uses during this period[1]. In order to understand the driving mechanism of historical LULCC in AMTR as a whole and in each subregion, we selected three first-level indicator layers: climate, topography, and human activities. LULC data in 2000 and 2020 were used to study the contribution of driving factors of historical LULCC. Slope

data were obtained from DEM in Arcgis10.8 (https://www.esri.com/en-us/arcgis /products/arcgis-desktop/resources), and Road distance was obtained using the Euclidean distance tool in Arcgis10.8. Finally, minimum temperature, maximum temperature, average temperature, solar radiation, Elevation, Slope, Night light, Population density, and road distance were selected. minimum temperature, maximum temperature, average temperature, and solar radiation 4 factors to represent climate, elevation, and slope to represent topography, night light, population density, and road distance to represent Human activity (S Fig 1), and then unify the resolution, row, and column number and coordinate system in Arcgis10.8, ready to run in PLUS models, all data is resampled to 1km resolution.

S Table 1. The PLUS model uses data.

Driving force	Driving factors	Unit	Resolution
**Climate**	Temperatur	℃	1km
	precipitation	mm	1km
	wind speed	$m \cdot s^{-1}$	1km
	solar radiation	$kJ \cdot m^{-2} \cdot day^{-1}$	1km
**Topography**	Elevation	m	30m
	Slope	°	30m
**Human activity**	Night light	-	450m
	Population density	km^{-2}	1km
	Road distance	m	1km

S4 Construction of ecological resistance surface and extraction of ecological corridor

Ecological processes (such as species migration) can be hindered by natural conditions and human activities. Ecological resistance surfaces effectively reflect horizontal resistance near the surface. we considered several key factors: elevation, slope, land cover type, distance from water source, and distance from roads. Elevation affects temperature and precipitation patterns, influencing species distributions and habitat suitability. Slope impacts soil stability and water drainage, which are crucial for species habitats in mountainous regions. Different land cover types (forests, grasslands, urban areas) have varying degrees of permeability for species movement. Proximity to water sources is critical for many species' survival, influencing their distribution and movement patterns. Proximity to roads can negatively impact species by increasing mortality risk and habitat fragmentation. These factors were integrated into the Linkage Mapper toolbox to identify potential corridors for species migration. The minimum resistance model (MCR), which is Estimating habitat isolation in landscape planning based on Knaapen et al., has been widely used in the construction of species protection and ecological security patterns[2,3]. The model calculated the cost of species movement between different ecological sources, reflected the potential possibility and trend of species movement, and simulated the process of organisms crossing different landscape bases[4]. Ecological corridor is the carrier of energy and material flow between source areas and the key component to maintain the connectivity and integrity

of ecological processes between regions. The minimum cumulative resistance model is adopted to comprehensively consider the three factors of source, distance and landscape interface to calculate the cost of species movement between different source areas. The formula for selecting the path of minimum cumulative resistance as the ecological corridor is as follows:

$$MCR = f_{min} \sum_{j=n}^{i=m} D_{ij} \times R_i \quad (1)$$

In the formula, MCR is the minimum cumulative resistance value; D_{ij} is the spatial distance of species from source j to landscape unit i; R_i is the resistance coefficient of landscape unit i to species movement; f_{min} represents the positive correlation between minimum cumulative resistance and ecological processes.

S Table 2. Area of KBAs.

TM ecological source area	Area/ km^2	TA ecological source area	Area/ km^2
**TM-CAR-1**	104.37	**TA-CAR-1**	173.39
**TM-CAR-2**	76.13	**TA-CAR-2**	897.63
**TM-CAR-3**	54.03	**TA-CAR-3**	112.04
**TM-KAR-1**	87.75	**TA-CAR-4**	94.70
**TM-KAR-2**	47.15	**TA-KAR-1**	594.86
**TM-KAR-3**	116.65	**TA-MAR-1**	153.38
**TM-MAR-1**	367.14	**TA-MAR-2**	869.62
**TM-MAR-2**	359.77	**TA-RAR-1**	290.76
**TM-MAR-3**	201.38	**TA-RAR-2**	161.39
**TM-MAR-4**	114.19	**TA-RAR-3**	2654.21
**TM-MAR-5**	230.84	**TA-RAR-4**	570.86
**TM-MAR-6**	168.22	**TA-RAR-5**	254.75
**TM-MAR-7**	85.95	**Total**	6827.59
**TM-MAR-8**	55.26		
**TM-RAR-1**	78.59		
**TM-RAR-2**	83.50		
**TM-RAR-3**	213.65		
**TM-RAR-4**	14088.90		
**Total**	16533.48		

S Table 3 Resistance surface construction set.

Influence factor	Classification index	Resistance value	Weight
**elevation**	0~500	75	0.13
	500~1500	10	
	1500~3500	1	
	3500~4500	10	
	> 4500	75	

**slope**	< 15	1	0.13
	15~25	10	
	25~35	50	
	34~45	75	
	> 45	100	
**landscape**	Cropland	50	0.32
	Forest	1	
	Grass	1	
	Shrub	1	
	Wetland	50	
	Water	500	
	Artificial surface	500	
	Bareland	100	
	Ice/Snow	100	
**Distance from water source**	0-6	1	0.28
	6-14	10	
	14-28	50	
	28-48	75	
	> 48	100	
**distance from road**	0 - 3	100	0.14
	3 - 8	75	
	8 - 16	50	
	16 - 28	10	
	> 28	1	

Note: The analytic Hierarchy process (AHP) was used to determine the influence weight, CR=0.006 < 0.1, and passed the consistency test.

S Table 4. IUCN Classification Criteria.

Classification	Explanation
**Critically Endangered (CR)**	A species is classified as CR when it is facing an extremely high risk of extinction in the wild in the immediate future.
**Endangered (EN)**	A species is classified as EN when it is facing a very high risk of extinction in the wild in the near future.
**Vulnerable (VU)**	A species is classified as VU when it is facing a high risk of extinction in the wild in the medium-term future.
**Near Threatened (NT)**	Although NT species are not currently considered threatened, they are close to qualifying for a

	threatened category in the near future and require monitoring and conservation efforts to prevent further decline.
**Least Concern (LC)**	A species is classified as LC when it does not qualify for any of the above categories and is widespread and abundant.

Reference

1. Liang X, Guan Q, Clarke KC, Liu S, Wang B, Yao Y. Understanding the drivers of sustainable land expansion using a patch-generating land use simulation (PLUS) model: A case study in Wuhan, China. Computers, Environment and Urban Systems. 2021;85: 101569.

2. Xiong L, Li S, Tang G, Strobl J. Geomorphometry and terrain analysis: Data, methods, platforms and applications. Earth-Science Reviews. 2022; 104191.

3. Zhao XQ, Xu XH. Research on landscape ecological security pattern in a Eucalyptus introduced region based on biodiversity conservation. Russ J Ecol. 2015;46: 59–70. doi:10.1134/S106741361501018X

4. Zhang L, Li J. Identifying priority areas for biodiversity conservation based on Marxan and InVEST model. Landsc Ecol. 2022;37: 3043–3058. doi:10.1007/s10980-022-01547-0
